# Simple diameter measurement as predictor of liver volume and liver parenchymal disease

**DOI:** 10.1038/s41598-022-04825-8

**Published:** 2022-01-24

**Authors:** D. Seppelt, T. Ittermann, M. L. Kromrey, C. Kolb, C. vWahsen, P. Heiss, H. Völzke, R. T. Hoffmann, J. P. Kühn

**Affiliations:** 1 Institute and Policlinic for Diagnostic and Interventional Radiology, University Hospital Carl-Gustav-Carus, TU Dresden, Dresden, Germany; 2 Institute for Community Medicine, University Medicine Greifswald, Greifswald, Germany; 3 Department of Diagnostic Radiology and Neuroradiology, University Medicine Greifswald, Greifswald, Germany; 4 Department of Diagnostic Radiology, University Medical Center Regensburg, Regensburg, Germany

**Keywords:** Magnetic resonance imaging, Liver cirrhosis, Liver fibrosis

## Abstract

To investigate the accuracy of liver diameters for estimation of liver size and to evaluate their application as tool for assessment of parenchymal liver disease. In the course of a population-based study, (SHIP) one thousand nine hundred thirty-nine volunteers underwent magnetic resonance imaging (MRI) of the liver including 3D gradient echo MRI sequences. Maximum liver diameters were measured in cranio-caudal (CC), anterior–posterior (AP), medial–lateral (ML) orientation. Diameters were compared with true liver volume assessed by liver segmentation. Additionally, age-dependent reference values for diameters were defined. Finally, accuracy of liver diameters was assessed to discriminate volunteers with healthy livers and participants with parenchymal changes, measured by MRI and laboratory. Reference values of liver diameters within the healthy population (n = 886) were defined as follows (mean ± standard deviation, confidence interval CI in cm): CC 17.2 ± 2, CI 13.6/21.2; AP 15.8 ± 1.9, CI 12.6/19.8; ML 19.7 ± 2.3, CI 15.8/24.6. There was a poor correlation using linear regression between liver diameter and true liver volume; CC 0.393, AP 0.359; ML 0.137. The AP direction shows the best correlation to discriminate between healthy and pathologic liver changes; AUC 0.78; p < 0.001, CC AUC 0.53; p < 0.001 and ML AUC 0.52; p = 0.008. Measurement of liver diameter, especially in the anterior–posterior direction is a simple option to detect chronic liver disease but less suitable for prediction of liver volume.

## Introduction

The exact determination of liver size is requested in clinical routine, since changes of liver volume can provide information about changes of liver parenchyma and could therefore play an important role in therapeutic decisions. There are many diseases that can be reflected in a change of liver size such as fatty liver diseases, hepatitis and liver cirrhosis. In addition to the diagnostic value, the liver volume also provides a prognostic factor in liver cirrhosis^1^. Likewise, the determination of the liver size is essential in preoperative planning prior to liver resections and liver transplantation^2–4^, as well as antecedent to interventional therapy such as radioembolization. Especially in the context of liver transplants, the exact determination can have a significant influence on the determination of outcome by reducing the postoperative risk of liver failure due to small-for-size syndrome^5^. For this reason, the exact determination of the liver size is of greatest clinical relevance.

The determination of liver size can be performed with the use of various imaging techniques. According to current standards, the most accurate method is to determine the liver volume using manual segmentation based on cross-sectional imaging^6^, although semi-automatic and automatic methods are convincing with increasing accuracy^7–9^. Since this procedure is very time-consuming and requires increased technical effort, its implementation in everyday clinical practice is challenging. Therefore, it would be desirable if the liver size could be estimated based on simple diameter measurements. In the literature, the information on the reference values, based on the measurement of liver diameter is controversially discussed, especially in ultrasound^10^. According to general opinion, an extension of < 16 cm measured in the midclavicular line can be considered as normal, even though this has to be seen critically^11^.

Therefore, the purpose of the study was to investigate the feasibility of diameter measurements to estimate liver size by a comparison of diameters with true liver volume. The second aim is to evaluate the accuracy of simple diameter measurement to differentiate between healthy study participants and those with liver parenchymal disease.

## Material and methods

### Study population

Data were selected from the database of a population-based study in Northeast Germany (SHIP)^12,13^. All participants were volunteers randomly selected from the 213,057 residents of Western Pomerania and representing a predominantly Caucasian population. The study consists of two stratified groups: The basic study (SHIP-0, between 1997 and 2001) consisting of 6265 individuals aged 20–79 years and its 10-year follow-up (SHIP-2, between 2008 and 2011). Simultaneously, another 10,000 adults from the region were invited and studied for the baseline examination of a second cohort (SHIP-TREND). Both SHIP-TREND and SHIP-2 participants voluntarily had the opportunity to perform a whole-body MRI^14^. In addition, various clinical data and laboratory were obtained within the SHIP study.

Based on the MRI examinations, 1,939 participants (1018 women and 921 men, mean age of 52.8 ± 13.9 years, range 21–89 years) were included in our study. The local Ethics Committee approved the prospective study. Informed consent was obtained from all study participants.

All study participants were divided into two groups according to the presence or absence of liver parenchyma disease defined by MRI and laboratory data as follows:

A. healthy volunteers with no evidence for parenchymal liver diseases in MRI and no elevated liver enzymes;
B. participants with parenchymal liver diseases, based on imaging and/or with elevated liver enzymes.

### Image acquisition

Liver MRI was acquired using a 1.5 Tesla scanner (Avanto, Siemens Healthcare, Erlangen, Germany) and an 8-channel phased-array surface coil. The measurements of liver diameters were performed with a three echo chemical-shift-encoded MRI sequence. The detailed MRI examination protocol has already been published^14^. Sequence parameters were: TR/TE1/TE2/TE3 = 11/2.4/4.8/9.6 ms; flip angle = 10° with an axial slab during a single 19-s breath-hold to acquire 3D gradient echo data with 3-echoes and flyback readout gradient.

### Assessment of liver diameters and calculation of liver volume

**Figure 1:**
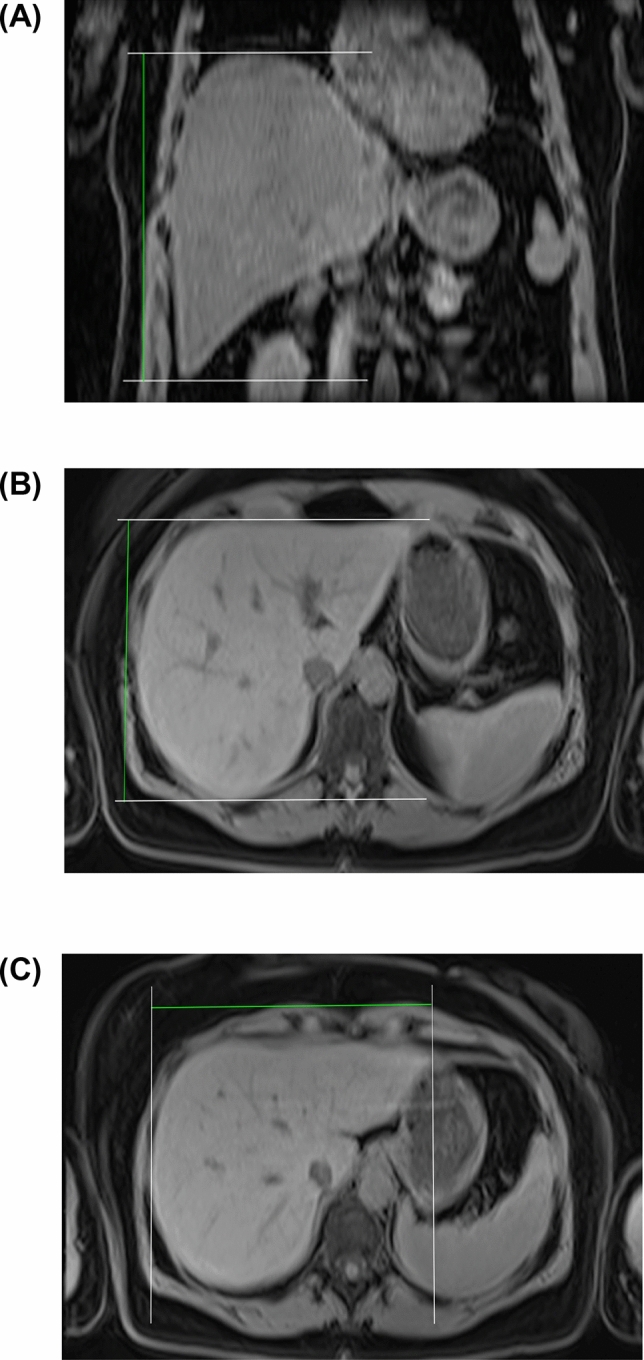
Sample measurement of diameters based on the MRI sectional images. Measurement of the maximum liver expansion in CC (**A**), AP (**B**) and ML (**C**) direction.

Assessment of liver diameters was determined using the software Horos (The Horos Project, sponsored by Nimble Co LLC d/b/a Purview, Annapolis, MD, USA). Measurements were obtained in maximum extension of the liver, more precisely in cranio-caudal (CC), anterior–posterior (AP) and medial–lateral (ML) orientation, by a trained student (VC) under supervision of an experienced radiologist (JPK) with more than 14 years of experience in MRI as described elsewhere^15^. In brief, maximum distances were determined by scrolling through the axial and coronal reconstructions to identify the position of the most cranial, caudal, anterior, posterior, lateral and medial part of the liver (Fig. 1).

Liver volume was assessed by semi-automatic segmentation based on the transversal MRI images with a 3 mm thickness using Voxar 3D (Version 4.2, Toshiba Medical Visualization Systems). For this purpose, the boundaries of the liver were defined manually by the user at intervals of more than 3 layers. The segmented parts of the liver were evaluated and corrected manually if necessary. The software calculated the liver volume automatically after complete segmentation. The segmentation was performed by an experienced radiologist with a professional experience of 6 years on the one hand, and independently by a trained radiology assistant after completing several measurements on training patients on the other hand.

### Assessment of liver parenchyma changes

For determination of liver parenchymal changes, chemical-shift-encoded MRI of the liver was used to analyze liver fat content (Proton-Density Fat Fraction, PDFF) as well as the liver iron content (Transverse Relaxation Rate, R2*), respectively. The algorithm for the creation of parametric maps has been described and published in detail elsewhere^15^. Measurements of liver fat content and liver iron content was performed by placing a ROI in the center of the liver using MRI-PDFF and R2* maps. As recommended by literature, the limit value for the presence of fatty liver was set to PDFF > 5.1%, and increased iron storage was evaluated from a measured value of > 41.0 s^−1^ in R2* maps^16^.

The evaluation of parenchymal liver diseases was assisted by measuring laboratory changes, in particular an increase in transaminases aspartate aminotransferase (ASAT), alanine aminotransferase (ALAT), and gamma-glutamyl transferase (GGT). Cut-off values were chosen as followed (based on the reference values of the local laboratory): 0.77 (ASAT), 0.59 (ASAT), 0.96 (GGT) µkatal/L.

### Statistical analysis

All data were presented as mean values including their standard deviation. The linear regression was used to compare the measurements of the diameters with the true liver volume regarding their significance for prediction of the true liver volume from one diameter in patients with and without liver disease.

The percentile rank was applied to the diameters both in the healthy population and in the group with liver parenchyma disease to calculate the mean distribution. Additionally, a gender-independent and a gender-dependent sub analysis were carried out. The same procedure was used to calculate the different liver volumes within the study populations.

Quantile regression was performed to show differences between percentiles of the diameters, as well as for the calculated volumes in relation to age and Body Mass Index (BMI). For each diameter and for the calculated volume, the reference values from the healthy group were determined by calculating the lower limit from the 2.5th percentile and the upper limit from the 97.5th percentile.

The accuracy of liver diameters was evaluated to distinguish volunteers with healthy liver and participants with parenchymal changes with the level of significance.

A statistical significance was considered in all evaluations with a P-value < 0.05. Statistical calculations were performed using Stata 15.1 (Stata Corporation, College Station, TX, USA).

## Results

Based on liver segmentation, the total liver volume over all study participants was 1623.6 ± 392.8 cm^3^. Measurement of liver diameters including subjects with and without liver diseases revealed the longest distance in ML direction; 19.8 ± 2.3 cm, followed by CC; 17.4 ± 2.1 cm, and AP; 16.9 ± 2.3 cm.

In the healthy population; the upper and lower limits of the diameters based on the 2.5th/97.5th percentile calculation for the gender-independent reference group resulted in AP direction were between 12.6–19.8 cm and the directions CC 13.6–21.2 cm and ML 15.8–24.6 cm, respectively. For the calculated liver volume, a reference value between 0.982–2.130 cm^3^ could be defined. A gender-specific subdivision showed that in men the individual diameters and the total liver volume were considerably larger, but a significant difference could only be found in the AP direction and in total liver volume. Results are shown as reference values defined by the 2.5th/97.5th percentile within the healthy population: AP (men 13.9/20.8 cm; women 12.4/18.1 cm; p < 0.001), CC (men 13.7/21.2 cm; women 13.6/21.2 cm; p = 0.203), ML direction (men 16.3/24.3 cm; women 15.6/24.7 cm; p = 0.405), total liver volume (men 1.127–2.406 cm^3^; women 0.945–1.989 cm^3^; p < 0.001).

Similar results can be found for the entire study population of participants with signs of liver alteration (AP; p < 0.001, CC; p = 0.559, ML; p = 0.023, total liver volume; p < 0.001), as well as in the gender-specific subdivision (AP; p < 0.001, CC; p = 0.052, ML; p = 0.005, total liver volume; p < 0.001).

The application of linear regression which includes all study participants, shows a weak correlation between the individual diameters and the total volume from liver segmentation, with the highest correlation in the CC (R^2^ = 0.393) and AP direction (R^2^ = 0.359) and the poorest correlation in medial–lateral direction (R^2^ = 0.137).

All mean diameters were significantly decreased in the healthy group compared to those with liver parenchyma diseases (Table 1). A significant difference in the evaluation of the individual diameters could be observed in all directions with a highly significant difference in AP and CC direction (p < 0.001) and a very significant result in ML direction (p = 0.008).

**Table 1:** Overview of study results, divided in whole population and healthy / pathologic subpopulation.

Diameter	AP (cm)	CC (cm)	ML (cm)	Volume (cm^3^)
Whole study population	16.9 ± 2.3	17.4 ± 2.1	19.9 ± 2.3	1623.6 ± 392.8
Study population with liver parenchyma changes	18.0 ± 2.1	17.6 ± 2.2	19.9 ± 2.3	1753.5 ± 41.5
Study population without liver parenchyma changes	15.8 ± 1.9	17.2 ± 2	19.7 ± 2.3	1469.3 ± 292.9

Overall, the AP diameter seems to have the highest significance with regard to the gender-independent differentiation between a healthy liver and liver changes. The ROC analysis yielded the following results in the calculation of the AUC (Table 2).

**Table 2:** Overview of ROC analysis, divided in whole population and gender specific subpopulation.

Diameter	ROC area	Std. Err	95% Conf. interval
**Whole population:**			
CC	0.53	0.013	0.50490–0.55623
AP	0.78	0.010	0.75446–0.79550
ML	0.52	0.013	0.48925–0.54129
**Men:**			
CC	0.50	0.020	0.46478–0.54387
AP	0.76	0.017	0.72225–0.78867
ML	0.55	0.02	0.51129–0.59331
**Women:**			
CC	0.56	0.018	0.51899–0.59126
AP	0.71	0.016	0.68210–0.74635
ML	0.53	0.018	0.49149–0.56333

In addition to the mean AP diameter of 15.8 ± 1.8 cm in the healthy study population, there was a significant gender difference with increased diameter in males of 17.2 ± 1.8 cm versus females of 15.1 ± 1.5 cm (p < 0.001).

**Figure 2:**
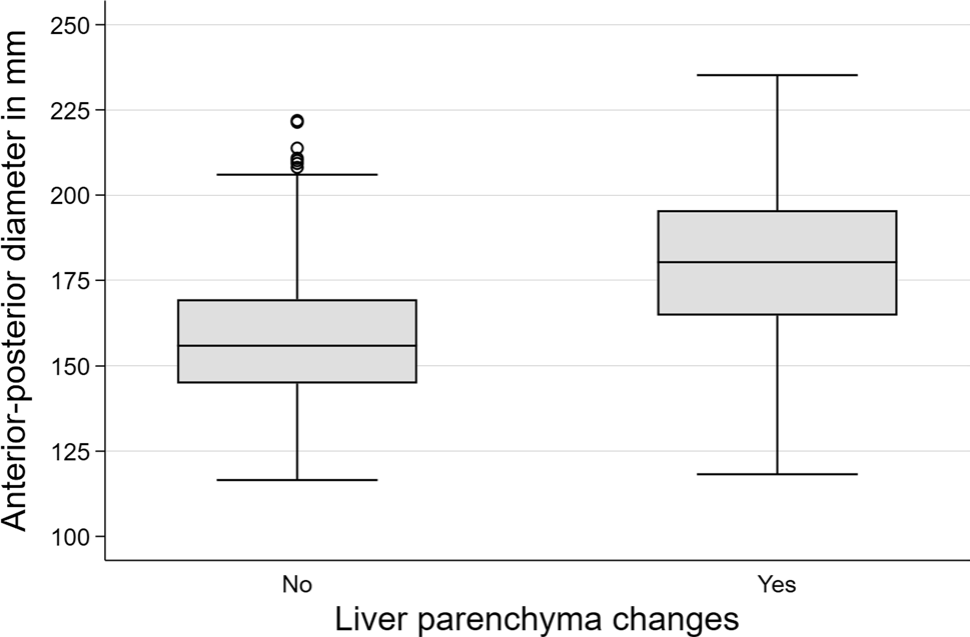
Overview of maximum AP diameter in patients with and without liver parenchymal changes.

Regarding the definition of a cut-off value for the detection of liver parenchymal changes, the intersection of the 75th percentile of healthy versus the 25th percentile of the group with liver parenchymal changes could be found at 17 cm maximum AP diameter (Fig. 2).

The mean and standard deviation of the calculated liver volumes showed a significant difference with a total volume of 1469.3 ± 292.9 cm^3^ in the healthy and 1753.5 ± 41.5 cm^3^ in the group with liver parenchyma changes (p < 0.001).

**Figure 3:**
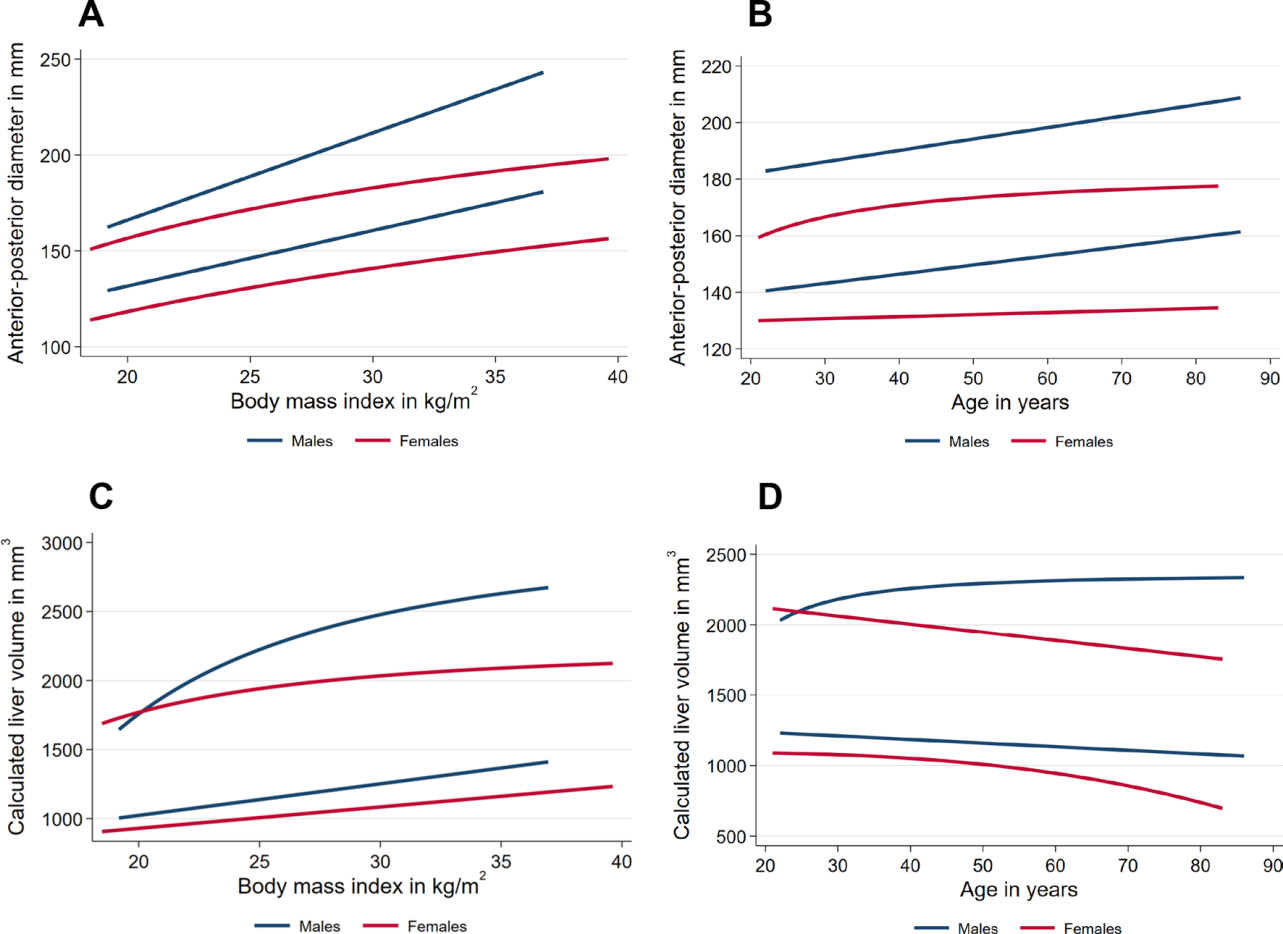
Correlation of AP diameter and the calculated liver volume with increasing BMI (**A**,**C**) and age (**B**,**D**). Percentiles of AP diameter (percentiles 5, 25, 50, 75, 95) by BMI and Age for men (blue) and women (red).

Regarding the development of the AP diameter with raising age and BMI, quantile regression showed a mainly significant difference for both, between the different percentiles. Similar results can be found in the observation of the development of liver volume with respect to increasing age and BMI, with the exception of the male group in which no significant development of liver volume with increasing age could be determined (Table 3). Overall, a steady increase of the AP-diameter with increasing age and BMI as well as of the total liver volume with increasing BMI could be observed. However, liver volume seems to decrease with increasing age (Fig. 3).

**Table 3:** Results of quantile regression for gender specific correlation of AP diameter and the calculated liver volume with increased BMI and age.

	Percentile of the AP diameter				
	5	25	50	75	95
**Men**					
Age	0.002	< 0.001	< 0.001	< 0.001	0.001
BMI	< 0.001	< 0.001	< 0.001	< 0.001	< 0.001
**Women**					
Age	0.133	0.021	< 0.001	< 0.001	0.002
BMI	< 0.001	< 0.001	< 0.001	< 0.001	< 0.001

	Percentile of the calculated liver volume				
	5	25	50	75	95
**Men**					
Age	0.679	0.057	0.039	0.020	0.443
BMI	0.002	< 0.001	< 0.001	< 0.001	0.003
**Women**					
Age	0.005	< 0.001	< 0.001	< 0.001	0.015
BMI	< 0.001	< 0.001	< 0.001	< 0.001	0.005

## Discussion

In our study, we evaluated the ability to determinate liver volume by using simple diameters. Additionally, the aim of the study was to analyze the predictive value of liver pathologies based on diameter or liver volume measurements. Apart from that we established reference values for liver diameters and corresponding cut-off values to discriminate volunteers and patients with liver parenchymal changes. Unfortunately, the results of our study demonstrated that it is not feasible to predict liver volume based on one measured diameter. This is in line with the existing literature, as several approaches for the determination of liver volume based on diametric measurements have already been described, which are always based on a composition of several diameters.

Our study results demonstrated that on the basis of a diameter measurement we can differentiate between subjects with a healthy liver and those with potential liver disease. We found the most appropriate diameter to differentiate between healthy and diseased liver by measuring the maximum AP direction.

In clinical routine, it is practical to determine the size of the liver based on diametric measurements. For this reason, the most important finding for clinical practice is that an AP of 17 or more means an enlarged liver and is associated with pathologies such as fatty liver and fibrosis. Therefore, in our opinion, such a measurement of the diameter should be implemented in the general diagnostics.

There are many approaches that are mainly based on sonography^11,17^, but can also be used for cross-sectional imaging^18^. In the literature, the information on the reference value is controversially discussed. According to general opinion, a diameter of < 16 cm measured in midclavicular line in CC direction can be regarded as normal, even if this was not sufficiently proven^11,19^. This limit value is also often used for measurements in AP direction^11^. The results of this study support this view of establishing a cutoff value of 17 cm for normal liver size. In contrast to existing literature, in which the measurement should be taken in the midclavicular line, our results indicate using the longest diameter in the AP extension. The remaining diameters do not show sufficient correlation with liver parenchymal changes based on our results, which is in contrast to existing studies^19^. This may be due to the fact that most studies to establish a reference value are methodologically based on measurements by ultrasound. In addition, our data suggest that gender differentiation in sizing makes sense, as men have a significantly larger liver than women. In this respect, the establishment of one gender-specific reference value each seems reasonable and appropriate.

However, these recommendations can only be transferred to ultrasound applications to a limited extent as Childs et al. could show that the most reliable measurements in ultrasound are made in the midclavicular line in AP and CC direction for the right hepatic lobe and in AP direction for the left hepatic^17^. Regarding the total volume of the liver, however, the authors consider the measurement in maximum AP direction to be sufficiently accurate, which supports the statement of our work as well as the transferability to measurements around ultrasound.

A conclusion on liver volume from a single measurement of the diameter cannot be drawn in any of these studies, which also supports our findings that the measurement of one diameter is not suitable for determining liver volume. Contrary to clinically common opinion, our results clearly show that the determination of the diameter of the liver alone only inadequately reflects its actual size. In the event that liver size needs to be determined accurately, other imaging modalities (e.g., CT or MRI) should be used.

Nevertheless, the measurement of a diameter seems to be suitable to differentiate between a healthy liver and the presence of liver diseases. Compared to the liver size of the healthy population, liver diseases show an enlargement of the liver, both in steatosis, fibrosis and iron overload. Against this background, the formation of reference values in a simple measuring procedure plays an important role in clinical diagnostics and becomes particularly relevant in the early detection of liver diseases.

This development of liver volume can be found irrespective of gender. The results of our study indicate that in the context of liver parenchymal changes there is an increase in liver size. This correlation is discussed in the literature regarding different parenchymal changes and in line with our results, which showed a significant increase in liver volume in relation of liver fibrosis^20,21^ and fatty liver disease^22^.

In accordance with previous literature by Kratzer et al.^19^ and Patzek et al.^23^, our study also showed a correlation between liver size and sex with a significantly higher liver volume in men compared to women. However, the study by Choukèr et al.^24^, in which no significant difference between liver size and sex in middle age could be found, also contradicts this. This could be due to differences in methodology, as liver size is calculated based on liver weight after autopsy, which may lead to inaccuracies in the derivation of size due to other physical changes.

Regarding the development of liver size with increasing age, the context is discussed controversially. Our data show an increase in liver volume in both men and women. This contrasts with earlier studies by Niederau et al.^25^ based on ultrasound examinations and De la Grandmaison et al.^26^ based on results from autopsy in which a reduction in liver size with increasing age was observed. One possible reason for the discrepancy in results here is the different methodological approaches as well as the small number within the study population. However, these results could already not be confirmed in subsequent studies such as that of Kratzer et al.^19^ and Patzak et al.^23^ were a significant correlation between changes in liver size and age was found. On the other hand, Özmen et al.^10^ found a statistically significant correlation between age and liver size in women and across the entire study population, although no correlation was found in the male group. Here, one reason for the different results can be found in the fact that the study by Özmen et al. was a population-based study in North Anatolian. These differences in origin may play a significant role in the present of discrepant results. Thus, interestingly, our results can only be applied to groups of different nationalities to a limited extent. Overall, in our opinion, a consideration of liver size with respect to age in a clinical context seems reasonable.

Regarding the relationship between liver size and weight, the relationship found in our study coincides with previous results from studies by Safak et al.^27^ and Toukan et al.^28^. However, in contrast to the study by Kratzer et al.^19^, we could not demonstrate a relationship between changes in liver size and body size, which may be due to the fact that here only the size of one diameter was compared in correlation to the body size, which seems to be a rather inaccurate method according to the data of our study and the consideration of only one diameter.

To the best of our knowledge this is the first study with the aim of establishing a reference value for liver diameters and volumes within the healthy Caucasian population as well as among residents with proven liver diseases. In our opinion, the diameters established within the study are a suitable tool for quickly diagnosing possible liver diseases and should therefore be used more frequently in clinical practice after imaging, especially MRI. This can play a role in clinical routine, as the liver is often included in the imaging of other diseases. Thus, liver changes can be detected early by measuring the AP-diameter even without symptoms. In our opinion, there would also be no reason why the data should not be transferred to CT imaging, which is the more widely used method. Another field of application could be in the therapy control of liver changes, for example in the context of iron overload or fatty liver changes, and thus offer the possibility of therapy changes.

The strength of this study lies in the large number of study participants on whose basis our data were collected.

There are some limitations in our study. First, the reference values of the liver were only collected based on MRI examinations. However, in reviewing the existing literature, we are of the opinion that these results can also be transferred to other imaging methods such as CT or ultrasound. Since this is a population-based study within the Caucasian population, it is questionable to what extent our results can also be transferred to other populations, especially against the background that Chandramohan et al.^29^ could show that population-based data cannot be directly transferred to other populations. Furthermore, due to the study design, the liver diseases could only be diagnosed based on the patient data and the image morphologic changes in the MRI and laboratory, a confirmation by biopsy as the standard of reference was not performed.

In summary, the measurement of one liver diameter is a fast and simple method to identify patients with parenchymal liver pathologies. The collection of the reference values for the diameter of the liver is particularly helpful as they are dependent on sex, age and BMI of the patient and thus allow a more precise differentiation. In general, a maximum diameter in AP direction of < 17 cm can be regarded as normal. The measurements should be carried out in the AP direction. Unfortunately, however, it is not possible to draw conclusions about the actual liver volume on the basis of the change of one diameter only.
